# Embedding research education and scholarly project development into a medical education curriculum: a transferable glocalized curriculum model

**DOI:** 10.3389/fmed.2026.1951481

**Published:** 2026-09-18

**Authors:** Cordelle Lazare, Leslie S. Craig, Rashida Daisley, Carlene Radix, Rhonda McIntyre

**Affiliations:** Community Medicine & Global Health, Office of External Affairs, Ross University School of Medicine, Bridgetown, Barbados

**Keywords:** curriculum innovation, experiential learning, glocalization, medical education, residency competitiveness, scholarly activity

## Abstract

Early experiential learning, including participation in research and community-engaged health promotion, fosters critical thinking, scholarly development, and socially responsive practice; however, these opportunities remain inconsistently integrated into pre-clinical curricula. Dense foundational science content, limited faculty mentorship capacity, and reliance on optional engagement contribute to inequitable access, with participation often limited to higher-achieving students. These gaps challenge compliance with accreditation expectations that require medical schools to provide students with opportunities for scholarly activity and faculty mentorship. Concurrently, the transition of United States Medical Licensing Examination Step 1 to pass/fail has increased the importance of scholarly productivity in residency selection, underscoring the need for scalable and equitable curricular models. Ross University School of Medicine delivers a longitudinal Community Medicine curriculum for all students, anchored by the Community-Based Assignment (CBA). The 12-credit Community Medicine program combines over 140 h of didactic, experiential, and active learning, with community-based clinical experiences, interprofessional collaboration, and reflective exercises to develop scholarly and clinical competencies. Within the CBA, students work in small groups to design and implement either a research project or health promotion intervention across three semesters. The model is scaffolded across conceptualization, implementation, and reflection, supported by training in epidemiology, biostatistics, ethics, and dissemination, alongside structured faculty mentorship. The model adopts a glocal approach by engaging students in addressing locally relevant health priorities within Barbadian communities while developing transferable competencies in social accountability, evidence-based practice, and community engagement that are applicable across diverse healthcare systems and global contexts. Between January 2023 to May 2026, students initiated 170 projects (106 research; 64 health promotion), with 49 ongoing and 16 having received IRB approval, reflecting sustained engagement and competency development in inquiry, collaboration, and community engagement. Anecdotal feedback highlights transformative learning, improved understanding of social determinants, and greater confidence in applying evidence-based practice. Future directions include implementing a structured project transfer mechanism to ensure continuity across cohorts and expanding faculty involvement. Increased integration of interdisciplinary and clinical faculty will strengthen mentorship, promote longitudinal engagement, and enhance scholarly productivity across the medical education continuum.

## Introduction

Early experiential learning in medical school is increasingly recognized for its long-term professional value (1, 2). Engagement in research and health promotion activities cultivates critical thinking, strengthens evidence-based practice, and supports the development of both physician-scientists and socially responsive clinicians (3). Research experiences enable students to generate and critically appraise knowledge while health promotion activities translate evidence into practice. Together, these experiences reinforce competencies in inquiry, analysis, collaboration, and community engagement, particularly when embedded within structured, institutional programs.

Despite these well-established benefits, integration of structured scholarly activity into the formal medical curriculum remains variable across medical schools, particularly during the pre-clinical years (4–6). Pre-clinical curricula are dominated by dense foundational science content required for progression to clinical training, constraining opportunities for research and health promotion engagement. Consequently, scholarly activities are treated as optional and primarily pursued by higher-achieving students, reinforcing inequities in access. Students with limited experience rely heavily on faculty mentorship and structured guidance to participate meaningfully in scholarly work (7). Yet, competing faculty demands and limited protected time restrict sustained mentorship, further diminishing student opportunities.

These challenges are particularly salient in the context of accreditation and evolving expectations for residency selection. Accreditation bodies, including the Liaison Committee on Medical Education (LCME) and Caribbean Accreditation Authority for Education in Medicine and Other Health Professions (CAAM-HP), require that students have access to scholarly activities and mentorship; however, inconsistent implementation may place programs at risk of non-compliance.

Beyond accreditation and workforce expectations, medical education is increasingly challenged to balance globally endorsed educational standards with the realities of local health systems and community needs. This tension has given rise to growing interest in glocalization, the deliberate adaptation of global educational frameworks to local sociocultural, epidemiological, and resource contexts (8–10). Within medical education, glocalization has been proposed as a strategy for ensuring that future physicians develop internationally recognized competencies while remaining responsive to the priorities of the communities they serve. However, practical examples of how glocalization can be operationalized within pre-clinical medical curricula remain limited, particularly in settings characterized by transnational student populations and diverse community contexts (9).

At the same time, an increasingly competitive residency landscape has heightened the importance of robust academic portfolios (11). The transition of United States Medical Licensing Examination (USMLE) Step 1 to pass/fail has further shifted emphasis towards alternative distinguishing attributes, with scholarly productivity emerging as an important indicator of applicant strength and preparedness (11–13).

Given these overlapping constraints, scalable and equitable solutions are urgently needed. Although innovative, student-led and elective initiatives have expanded research opportunities, access remains varied and often contingent on institutional resources, faculty capacity, or students’ ability to participate outside the formal curriculum (4, 7, 14). Consequently, there remains a need for practical models that ensure equitable access to research training while integrating scholarship into core medical education. In response, we present a transferable model that embeds research as a required curricular component of medical education, intentionally integrating mentorship, scholarship, and research engagement into the formal curriculum rather than relying primarily on optional or extracurricular activities. By ensuring that all students participate in the research process, the model promotes the development of critical inquiry, evidence-based practice, and scholarly dissemination skills while reducing reliance on extracurricular engagement. It also offers a practical approach for medical schools seeking to systematically integrate research training into required curricular experiences. The model further operationalizes principles of glocalization by engaging students in addressing locally relevant community health priorities while developing globally transferable competencies in research, social accountability, evidence-based practice, and community engagement. Situated within an international medical education context, the approach demonstrates how community-engaged scholarship can be aligned with broader global health perspectives and leveraged to prepare future physicians to navigate the social, cultural, and structural determinants of health across diverse populations and healthcare systems. Importantly, this model is adaptable to a range of institutional contexts and may assist medical schools seeking to expand meaningful research experiences, promote equitable access to scholarly activity, and make students more competitive for residency.

## Pedagogical framework

Ross University School of Medicine (RUSM) follows a Doctor of Medicine (MD) curriculum delivered across three semesters (spring, summer and fall) per academic year, with each semester spanning 16 weeks. The pre-clinical curriculum extends across five semesters and utilizes an integrated curriculum across the foundational medical sciences, clinical foundations, professional development, and community-engaged service learning. Within this broader pre-clinical curriculum, the Community Medicine (CM) curricular thread is embedded.

RUSM launched the 12-credit CM curricular thread in January 2023 to prepare students to become competitive, community-oriented physicians and healthcare leaders equipped to address primary care needs and social determinants of health (SDoH) across local and global contexts.

The CM thread is a longitudinal component of the broader pre-clinical curriculum that spans five semesters and includes over 140 h of structured content, delivered via complementary and mutually reinforcing modalities, including didactic instruction, active learning strategies, experiential community-engaged service learning, reflective exercises, and interprofessional collaboration (Table 1). Rather than functioning as a stand-alone course, the thread is intentionally woven throughout the pre-clinical curriculum to provide progressive opportunities for students to develop competencies in community health, research, health promotion, social accountability, and interprofessional collaboration. These approaches are intentionally integrated. For example, reflective exercises build on experiential clinical encounters [called community clinical experiences (CCEs)], while active learning and collaborative experiences reinforce the role of interdisciplinary partnerships and community engagement in research, health promotion, and patient-centered care. CCEs, comprising ten structured clinical engagements, provide hands-on exposure to community-based practice, while also enabling clinical skills development, professional identity formation, and identification of community health priorities. Further, these encounters serve as catalysts for developing research questions and health promotion initiatives.

**Table 1:** Integrated curricular design linking learning modalities to intended research, social accountability and scholarly competencies.

Curricular design elements	Didactic learning	Active learning	Experiential community-engaged service learning	Reflective learning	Inter-professional learning
Illustrative curricular components	Enhancement sessions (live and recorded lectures)	Mandatory team-based learning (TBL) sessions and workshops	10 community clinical experiences (CCEs)	Structured reflective exercises (post-CCEs)	Inter-professional education (IPE) workshops; Community-based assignments (CBAs)
Intended competencies	Foundational knowledge in research design, epidemiology, and public health	Clinical reasoning, collaborative problem-solving, and applied research skills	Recognition of social determinants of health and health system inequities	Reflective exercises, bias identification, and professional identity formation	Interdisciplinary communication, team-based competencies, and appreciation of diverse professional roles

Didactic and experiential components in semesters 1 and 2 prepare students for the Community-Based Assignment (CBA) through an embedded scholarly program that provides focused training in research and health promotion methodology. Core content includes epidemiology, biostatistics, study design, and critical appraisal, alongside health promotion principles such as needs assessment, intervention design, implementation, and evaluation. Practical sessions emphasize skill development in research question formulation, protocol development, data collection and analysis, ethical conduct, institutional review board (IRB) processes, and scholarly writing and dissemination. Moreover, research ethics training is completed by all students in semester 2.

The pedagogical design of the CM thread and preparation for the CBA is grounded in several complementary educational theories and evidence-based practices. The curriculum is informed by Kolb's Experiential Learning Theory, which postulates that learning occurs through a cyclical process of concrete experience, reflective observation, abstract conceptualization, and active experimentation (15). Through CCEs, students engage directly with patients and communities, reflect on these encounters, integrate new knowledge with foundational concepts, and subsequently apply their learning through research and health promotion projects. Experiential learning has widely been recognized as an effective approach for developing clinical reasoning, professional identity, and practice competencies in medical education (16). The integration of CCEs, reflective exercises, and scholarly activities with the CM curriculum reflects this experiential learning cycle and enables students to connect theoretical concepts with authentic community health challenges.

The curriculum is also informed by the social constructivist learning theory, which emphasizes that knowledge is constructed through interaction, dialogue, and collaborative problem-solving (17). Students work in small groups, engage with faculty mentors, community stakeholders, and interprofessional partners, and participate in team-based learning activities that support the construction of knowledge and the development of shared understanding of community health issues. Similarly, situated learning theory and the concept of communities of practice highlight the importance of learning within authentic social and professional contexts. By embedding learning within community settings and exposing students to real-world healthcare challenges, the curriculum promotes meaningful participation in the practice of public health, primary care, and community medicine.

The CBA further incorporates principles of scaffolding and constructive alignment. Learning activities are intentionally sequenced across semesters to facilitate progressive competency development beginning with foundational instruction in epidemiology, biostatistics, critical appraisal, and research methods, followed by supervised application through project development and implementation. This scaffolded approach supports the gradual transfer of responsibility from faculty to learners while ensuring alignment between learning objectives, instructional activities, and assessment strategies (18). Such longitudinal and developmental approaches have been associated with improved learning engagement, deeper learning, and enhanced retention of knowledge and skills.

Furthermore, the curriculum draws on the principles of service-learning, defined as a structured educational approach that combines meaningful community engagement with guided reflection to enhance learning and civic responsibility. Community partnership, health promotion initiatives, and needs-driven research projects enable students to address authentic community priorities while developing competencies in social accountability, cultural humility, advocacy, and population health. Evidence suggests that service-learning experiences contribute positively to students’ understanding of social determinants of health, professional values, and commitment to underserved populations (19).

## Learning environment (setting, students, faculty); learning objectives; pedagogical format

RUSM is a CAAM-HP accredited international medical school (IMS) located in Barbados, dedicated to training future physicians through an innovative, student-centered, and experiential curriculum. The four-year MD program requires students to complete a total of 170 academic credits to qualify for graduation. Of these, 80 credits are earned during the pre-clinical phase of the curriculum in Barbados, where students engage in foundational medical sciences and early clinical learning experiences. The remaining 90 credits are completed during the clinical sciences phase in the United States through affiliated teaching hospitals and healthcare institutions. RUSM serves a full-time student population, largely from the United States and North America with a smaller number of students from international locations, and delivers a medical education designed to develop ethical, compassionate, patient-centered, and culturally competent physicians committed to improving health outcomes in diverse local and global communities. Through the integration of experiential learning opportunities across both pre-clinical and clinical training, the curriculum prepares graduates for the complex demands of contemporary medical practice.

At RUSM, the CM curricular thread of the pre-clinical curriculum culminates in the CBA, a mandatory, longitudinal, scaffolded learning experience that is embedded across three pre-clinical semesters (semesters 3–5). Working collaboratively in in-person small groups (i.e., 6–7 students), learners design and implement either a research project addressing a specific community health issue or a health promotion intervention aimed at improving community well-being. The CBA learning objectives are to analyze community health needs and identify specific issues that can be addressed through research or health promotion; design and create a comprehensive research or interventional proposal, including problem statement, goals, objectives, and methodology; implement and execute a research project or health promotion initiative, apply theoretical knowledge to practical scenarios; evaluate the effectiveness of the project or intervention using appropriate metrics and assess the outcomes; communicate findings and progress through regular updates and reflect on the overall project experience and learning.

Both pathways emphasize inquiry, application, and the development of scholarly competencies. Further, across both pathways, the CBA progresses through three stages: conceptualization, implementation, and reflection (Figure 1). In semester 3, student groups define a problem and develop a proposal; research groups focus on methodology, feasibility, budgeting, and timelines while health promotion groups design an evidence-based intervention plan, including implementation and monitoring strategies. In semester 4, research groups complete an IRB application and submit a progress report, while health promotion groups initiate implementation and provide updates. By semester 5, all groups complete project activities, engage in structured reflection, and submit final reports summarizing their work and output. Students receive iterative feedback and faculty mentorship to ensure methodological rigor and ethical alignment. Dedicated CM faculty provide guidance on study or intervention design, community engagement, ethical considerations, data interpretation and dissemination. Regular faculty-student meetings, at least once per semester, support sustained engagement, skill development, and continuous improvement in project quality. Beyond the formal curriculum, RUSM hosts three annual in-person symposia that enable students to disseminate their scholarly work and strengthen their engagement in academic scholarship. These scholarly events bring students, faculty, staff, and invited collaborators to showcase research projects and health promotion initiatives addressing local and global health challenges. Presentations include project proposals, work in progress, completed research, and health promotion interventions, providing students with formative feedback, opportunities for scholarly communication, and exposure to interdisciplinary perspectives. The symposia also serve as a platform for mentorship, collaboration, and recognition of student scholarship.

**Figure 1:**
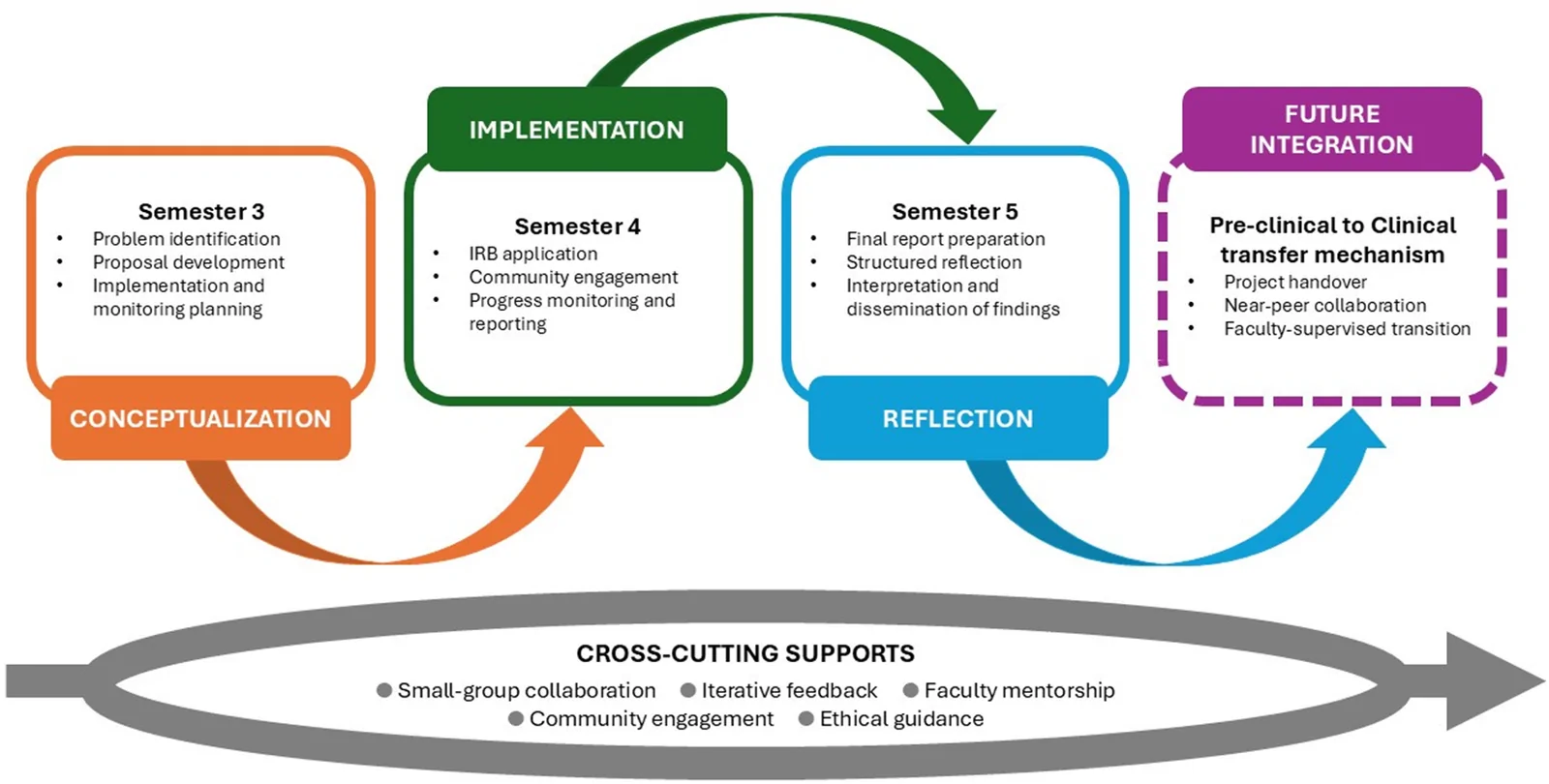
Longitudinal community-based assignment (CBA) implementation model.

Ethical research practice and community engagement are core components of the CBA model. Research projects involving human participants require formal IRB review, and students are guided through the preparation of protocols, consent procedures, and data protection plans, strengthening competencies in ethical and responsible research conduct. The CBA also fosters collaboration with local community stakeholders, including public health organizations and government agencies, which enhances project feasibility, ensures cultural relevance, and aligns student-led initiatives with priority health needs of local Barbadian communities. This approach reinforces principles of social accountability and situates learning within authentic settings (20, 21).

Mandatory participation ensures equitable access to experiential learning and scholarly development. Students build competencies in research design, health promotion, ethics, and community-based practices. Successful fulfillment of the CBA curricular requirements contributes to the awarding of a Certificate in Community Medicine alongside the MD degree. Projects developed through the CBA are assessed primarily on the quality of project conception, planning, methodological rigor, and feasibility rather than on successful implementation. Given the duration of the five-semester pre-clinical curriculum, the program emphasizes the development of well-designed, evidence-informed projects that can address identified community needs. Although implementation is strongly encouraged and supported whenever feasible, students are not penalized if circumstances prevent project implementation during their time in Barbados, provided that all required project planning and development components have been satisfactorily completed.

## Results to date

Since the inception of the CBA, through May 2026, 170 student-led projects have been developed, including 106 research and 64 health promotion projects. Of these, eight projects have been implemented (one research project and seven health promotion interventions), 113 are archived (75 research projects and 38 health promotion interventions), and 49 are ongoing (30 research projects and 19 health promotion interventions). Implemented projects are those that progressed beyond the planning phase and were executed within the intended community or target population during the students’ pre-clinical training, while archived projects represent those that were determined to be feasible and met all curricular requirements for project development and planning but were not implemented during the students’ pre-clinical training. Ongoing projects refer to initiatives that remain under active development by pre-clinical students. Additionally, 16 research projects have received IRB approval to date, representing a critical milestone in students’ scholarly development and underscoring the program's emphasis on rigorous academic engagement. While the number of archived projects remains high, participation in the CBA also provides students with opportunities to develop foundational research and health promotion competencies that support future scholarly engagement during the clinical sciences and throughout their professional careers. Moreover, the three annual symposia have showcased more than 80 student presentations since their inception in 2023.

Beyond these outputs, the distribution and progression of projects reflect the intentional design of the CM curriculum, particularly the CBA, as a scaffolded experiential learning platform that supports active student engagement in both the generation and application of evidence within authentic contexts. Experiential learning in medical education can promote a deeper understanding of SDoH, enhance critical thinking, and foster personal and professional growth through real-world engagement, while also strengthening competencies in communication, collaboration, and professionalism.

Within this context, the CBA provides students with structured opportunities to move beyond passive knowledge acquisition to active participation in scholarly inquiry and community engagement. By designing and conducting projects that address locally relevant health issues, students develop transferable skills in problem identification, stakeholder engagement, implementation, and evaluation, while also cultivating a sense of social accountability and responsiveness to community health priorities (22, 23). Importantly, such experiences have been associated with transformative learning, shaping students’ perspectives on future roles, and reinforcing interest in careers that integrate clinical practice with population health and community engagement (1). Collectively, these outcomes suggest that the CM curriculum not only strengthens students’ academic and professional competencies but also contributes to the development of reflective, community-oriented physicians equipped to address complex health challenges across diverse settings.

As part of our efforts to strengthen research literacy within the curriculum, early involvement in the IRB process provides students with a practical and nuanced understanding of ethical principles, regulatory frameworks, and the responsibilities inherent in conducting research involving human participants. This experience strengthens competencies in study design, risk assessment, and participant protection, while fostering a culture of ethical accountability and professionalism that is essential for future clinical and academic practice. Importantly, engagement with the full ethical review process, from protocol development to submission and revision, demystifies research governance and equips students with the practical skills and confidence needed to independently navigate complex regulatory environments in future scholarly endeavors.

Involvement in scholarly inquiry also strengthens critical thinking, analytical reasoning, and collaboration skills, with some students contributing to peer-reviewed publications and gaining confidence in engaging with evidence. Anecdotal student feedback further illustrates the perceived value of experiential community-engaged service learning within the CM curriculum. Students consistently describe these experiences as transformative, noting that direct engagement with community contexts deepens their understanding of the social and cultural determinants of health. Participation in the CBA helps students connect theoretical knowledge with real-world application, broadening perspectives beyond a purely biomedical model to better understand patient behavior and health outcomes. Students also report that these experiences foster a more reflective approach to patient care, helping them understand the underlying drivers of health decisions. Even for those not pursuing research careers, transferable skills such as inquiry, communication, and problem-solving enhance clinical practice. Overall, these experiences contribute to the development of socially responsive, well-rounded physicians.

## Discussion

The next phase of the CBA will prioritize expanded faculty development and implementation of a structured project transfer mechanism to strengthen continuity and early scholarly engagement. RUSM is broadening faculty involvement by implementing a framework that integrates interdisciplinary faculty as content experts, fostering faculty-student mentorship aligned with institutional goals for research capacity and publication readiness. Faculty development efforts will focus on strengthening competencies in project supervision, ethical guidance, and dissemination, reflecting expectations that faculty provide methodological support, advise on IRB requirements, and offer timely feedback throughout the project lifecycle.

Within the current model, student engagement in research and health promotion initiatives occurs alongside the demands of an intensive pre-clinical curriculum, with implications for the scope and feasibility of projects within the limited (semesters 3–5) timeframe. Specifically, while health promotion activities may be more readily implemented, research projects may be more susceptible to delays, including those associated with IRB processes (24). To address these constraints, a formalized CBA transfer mechanism has been developed, and will soon be implemented to support archived project continuity through structured handover and collaborative models, ensuring that projects initiated by students can be sustained as they transition to clinical sciences, with the involvement of pre-clinical students under faculty supervision (Figure 1). This approach will enable earlier exposure to scholarly activity, promote longitudinal skill development, cultivate a sustainable culture of scholarship across the medical education continuum, make students more competitive for residency matching, and create sustained student engagement in research and health promotion throughout clinical sciences. Importantly, participation in or adoption of an existing CBA-derived project does not replace the requirement for students to complete their own CBA at the designated point within the curriculum. To satisfy curricular requirements, all students must independently develop a project through the CBA framework, as no academic credit or curricular recognition is awarded for contributions to projects initiated by previous cohorts.

Beyond supporting the continuity of existing projects, there is a compelling rationale for integrating scholarly opportunities more broadly within the medical education curriculum. Contemporary physicians are expected not only to apply evidence-based medicine but also to critically evaluate, generate, and translate evidence into practice. Embedding structured and longitudinal scholarly experiences throughout medical education enables students to develop competencies in scientific inquiry, critical appraisal, data interpretation, scholarly writing, and research ethics, all of which are essential for lifelong learning and clinical decision-making. Expanded scholarly opportunities also promote intellectual curiosity, strengthen analytical and problem-solving skills, and foster a deeper understanding of population health and quality improvement. Integrating these opportunities within the curriculum, rather relying solely on extracurricular participation, ensures equitable access to scholarly experiences for all students and reinforces scholarly activity as a core professional competency alongside clinical knowledge, communication, and patient care skills.

As an IMS, an additional consideration is that pre-clinical students engage in these projects in Barbados prior to returning to the US for clinical rotations. This positioning creates a unique opportunity to advance a glocal model of medical education, whereby students address locally relevant community health priorities while developing knowledge, skills, and professional attributes that are transferable across diverse healthcare systems and populations. Through engagement with Barbadian communities, students gain firsthand experience in understanding how social, cultural, economic, and structural determinants influence health outcomes, while simultaneously developing competencies needed to address similar challenges in future clinical practice. Integrating clinical faculty into the projects of students who desire to continue their projects during clinical rotations will further strengthen this local-to-global continuum of learning, support mentorship, and enhance alignment with future practice. By maintaining continuity across the pre-clinical-clinical transition, the CBA will reinforce the application of evidence-based medicine in patient care while strengthening students’ capacity to contribute meaningfully to research, quality improvement, and community health initiatives across both local and global settings throughout their medical training.

The CBA model requires a combination of curricular, faculty, administrative, and community resources to support its successful implementation and sustainability. A dedicated curricular structure is essential, with protected time allocated for community needs assessments, project development, mentorship, and scholarly dissemination activities. Faculty resources should include educators with expertise in research methods, community engagement, public health, and health promotion who can provide longitudinal mentorship and oversight throughout the project lifecycle. Administrative infrastructure is also necessary to coordinate community partnerships, manage project tracking systems, facilitate ethics review processes where applicable, and support communication among students, faculty, and community stakeholders. Access to research support services, including library resources, data collection platforms, and training in research methodology and program planning, further enhances student success. Establishing and maintaining partnerships with community organizations is particularly important, as these collaborations provide the settings and stakeholder engagement needed for meaningful community-based scholarship. Notably, implementation of the CBA model may not require substantial financial investment because the primary focus is on project identification, development, planning, and feasibility assessment rather than project execution. Nonetheless, funding can indeed strengthen model implementation by supporting scholarly activities and outputs, including analytic software access and publication goals. A centralized repository for completed and ongoing projects can further strengthen program sustainability by facilitating project continuity, minimizing duplication of effort, and supporting the transfer of feasible projects across student cohorts. Consequently, institutions with limited resources can adopt the model provided there is institutional commitment to experiential learning, faculty mentorship, community engagement, and scholarly development.

## Acknowledgement of conceptual, methodological, environmental, or material constraints

Several constraints should be considered when interpreting and implementing the CBA model. Conceptually, the curriculum seeks to balance research, health promotion, community engagement, and scholarly dissemination within a limited pre-clinical timeframe, which may constrain the depth and scope of individual projects. Methodologically, research projects are subject to ethical review and regulatory requirements that can delay implementation and completion. Environmentally, project success depends on sustained community partnership, stakeholder engagement, and the availability of appropriate field settings, which may vary over time. Materially, the model relies on faculty mentorship capacity, institutional support, and student time within an already intensive medical curriculum. As an IMS, additional challenges arise from student transitions between pre-clinical training in Barbados and clinical rotations in the United States, which may affect project continuity. Despite these constraints, the structured, longitudinal design of the CBA provides a scalable framework for equitable participation in scholarly and community-engaged learning while supporting the development of socially accountable physicians.

## Data Availability

The original contributions presented in the study are included in the article/Supplementary Material, further inquiries can be directed to the corresponding author.
